# Relationship between the Balance of Hypertrophic/Hyperplastic Adipose Tissue Expansion and the Metabolic Profile in a High Glucocorticoids Model

**DOI:** 10.3390/nu8070410

**Published:** 2016-07-02

**Authors:** María Guillermina Zubiría, Ana Alzamendi, Griselda Moreno, Andrea Portales, Daniel Castrogiovanni, Eduardo Spinedi, Andrés Giovambattista

**Affiliations:** 1 Neuroendocrinology Laboratory, Multidisciplinary Institute of Cellular Biology (IMBICE, CICPBA-CONICET-UNLP), Calles 526 10 y 11, La Plata 1900, Argentina; gzubiria@imbice.gov.ar (M.G.Z.); aalzamendi@imbice.gov.ar (A.A.); andreaeportales@gmail.com (A.P.); dcastrogiovanni@yahoo.com (D.C.); 2 Biology Department, School of Exact Sciences, Universidad Nacional de La Plata, La Plata 1900, Argentina; 3 Institute of Immunological and Physiopathological Research (IIFP, CONICET-UNLP), School of Exact Sciences, Universidad Nacional de La Plata, La Plata 1900, Argentina; gmoreno@iifp.laplata-conicet.gov.ar; 4 Center of Experimental and Applied Endocrinology (CENEXA, UNLP-CONICET, PAHO/WHO Collaborating Center for Diabetes), La Plata Medical School, Universidad Nacional de La Plata, La Plata 1900, Argentina; spinedi@cenexa.org

**Keywords:** retroperitoneal adipose tissue, adipogenesis, stromal vascular fraction cells, cell determination, adipogenic competency

## Abstract

Adipose tissue (AT) expansion is the result of two processes: hyperplasia and hypertrophy; and both, directly or indirectly, depend on the adipogenic potential of adipocyte precursor cells (APCs). Glucocorticoids (GCs) have a potent stimulatory effect on terminal adipogenesis; while their effects on early stages of adipogenesis are largely unknown. In the present work, we study, in a model of high GC levels, the adipogenic potential of APCs from retroperitoneal AT (RPAT) and its relationship with RPAT mass expansion. We employed a model of hyper-adiposity (30- and 60-day-old rats) due to high endogenous GC levels induced by neonatal treatment with l-monosodium glutamate (MSG). We found that the RPAT APCs from 30-day-old MSG rats showed an increased adipogenic capacity, depending on the APCs’ competency, but not in their number. Analyses of RPAT adipocyte diameter revealed an increase in cell size, regardless of the rat age, indicating the prevalence of a hypertrophic process. Moreover, functional RPAT alterations worsened in 60-day-old rats, suggesting that the hyperplastic AT expansion found in 30-day-old animals might have a protective role. We conclude that GCs chronic excess affects APCs’ adipogenic capacity, modifying their competency. This change would modulate the hyperplastic/hypertrophic balance determining healthy or unhealthy RPAT expansion and, therefore, its functionality.

## 1. Introduction

Glucocorticoids (GCs) have several effects on adipose tissue (AT) biology: among others, they regulate terminal adipocyte differentiation, AT endocrine function and lipogenic-lipolysis balance [1,2,3]. Some of these effects are more predominant on visceral (VAT) rather than on subcutaneous (SCAT) AT function [4]. One clear example is the Cushing’s syndrome (CS) phenotype, mainly characterized by high GC levels in blood and increased VAT rather than SCAT mass [5]. Other features of CS, some of them shared by the human metabolic syndrome (MS) phenotype, are the presence of VAT hypertrophic adipocytes, altered lipid metabolism and impaired adipokine secretion [6]. The existence of these alterations suggests that GCs have a pivotal role in the pathogenesis of central obesity and the associated alterations seen in the CS phenotype.

Neonatal i.p. administration of the neurotoxin monosodium l-glutamate (MSG) in rodents causes neuronal loss, mainly at the hypothalamic arcuate nucleus level [7], triggering a failure in hypothalamus-pituitary-adrenal axis regulation, which results in high plasma GC levels [8]. Because of this reason, the MSG treatment has been widely used to generate animal models resembling that of the human CS phenotype. We have extensively used the MSG rat model, which is characterized by increased VAT mass with hypertrophic adipocytes and an altered peripheral pattern of adipokines [8,9]. 

It is generally accepted that the ability of an AT depot to expand its mass depends on two processes: the generation of new adipocytes (adipogenesis) and the hypertrophy of mature AT cells. It has been proposed [10] that at the AT level, hyperplasia contributes to maintaining a pool of small and functional adipocytes, thus preventing the development of metabolic alterations associated with hypertrophic obesity [11]. Indeed, adipocyte hypertrophy is associated with cell dysfunction, such as insulin resistance [12] and changes in the adipokine secretion pattern [13,14]. 

During the adipogenic process, adipose precursor cells (APCs) differentiate into mature adipocytes in two sequential steps: commitment of mesenchymal stem cells (MSCs) to APCs, acquiring their adipogenic potential and restricting them to the adipocyte linage, followed by terminal adipocyte differentiation [15]. CD34 is a cell surface antigen that distinguishes between adipogenic and non-adipogenic cell subpopulations in the stromal vascular fraction (SVF) of the AT [16]. This CD34^+^ subpopulation expresses almost exclusively the transcriptional factor Zinc finger protein 423 (Zfp423) [17], which in turn activates the basal expression of cell peroxisome proliferator-activated receptor (PPAR)-γ2, a key pro-adipogenic signal that assures APCs conversion into adipocytes [18]. The differential expression of both transcriptional factors determines the competency of APCs, that is, the cell’s ability to differentiate into adipocytes after the addition of defined stimuli [15].

GCs are one of the most important cell differentiation inducers through their inhibitory effect on Preadipocyte Factor 1 (Pref-1) and Wingless-type MMTV integration site family member 10b (Wnt-10b) expression [19,20,21]. Both, Pref-1 and Wnt-10b, are highly expressed in preadipocytes and absent in mature adipocytes [22,23]. In vitro experiments with the preadipocyte cell line 3T3-L1 have shown that Pref-1 is an early target for dexamethasone (DXM) action and that its expression decreases with high DXM concentrations, at the same time that adipocyte differentiation increases [19]. Similarly, in vivo and in vitro studies demonstrated that the inhibition of the Wnt/b-catenin signaling pathway by methylprednisolone or DXM, respectively, can promote adipocyte differentiation [21,24]. Several studies show that GCs can affect adipogenesis through their binding to mineralocorticoid (MR) and/or glucocorticoid (GR) receptors [25,26,27], although the contribution of MR and GR in mediating GCs’ adipogenic effect has not been fully understood. 

While the activity of GCs on adipocyte terminal differentiation has been extensively studied, the effect of chronic GC exposure of SVF cells remains almost unexplored. Using adult male MSG rats, it has been previously demonstrated that adipogenesis is inhibited as a consequence of long-term GC exposure of SVF cells, thus contributing to hypertrophic retroperitoneal AT (RPAT) mass expansion [28]. The present study focuses on the APCs’ adipogenic potential during in vivo RPAT mass expansion under an earlier chronic GCs circulating excess.

## 2. Materials and Methods

### 2.1. Animals and Treatment

Sprague-Dawley newborn pups were i.p. injected with either 4 mg/g of body weight (BW) MSG (Sigma Chemical CO., St. Louis, MO, USA) dissolved in sterile 0.9% NaCl or 10% NaCl (litter-mate controls; CTR) on alternate days between 2 and 10 days of age [9]. CTR and MSG male rats were used for experimentation at 30 days of age. Additional groups of rats at 60 days of age were used for a set of comparative experiments (SVF cell composition analysis by flow cytometry and adipocyte size measurements, as described below). Animals under non-fasting (basal) conditions (between 08:00 a.m. and 09:00 a.m.) were euthanized, and trunk blood was collected into Ethylenediaminetetraacetic acid (EDTA)-coated tubes. After centrifugation (3000 rpm for 15 min at 4 °C), plasma samples were kept frozen (−20 °C) until metabolite measurements. Retroperitoneal adipose tissue (RPAT) pads were aseptically dissected, placed into sterile conic tubes containing 10 mL sterile Dulbecco’s Modified Eagle’s Medium-Low Glucose (1 g/L) (DMEM) and immediately processed as describe below (Section 2.3). Animals were euthanized according to protocols for animal use, in agreement with the National Institutes of Health (NIH) Guidelines for the care and use of experimental animals. All experiments were approved by our Institutional Animal Care Committee (ID 03-05-12).

### 2.2. Plasma Measurements

Circulating levels of leptin (LEP), insulin (INS) and corticosterone (CORT) were determined by specific radioimmunoassays (RIAs) developed in our laboratory as previously validated and described [8]. LEP (standard curve 0.05–25 ng/mL) coefficients of variation (CV) intra- and inter-assay were 4%–7% and 9%–11%, respectively. INS (standard curve 0.08–10 ng/mL) CVs intra- and inter-assay were 3%–7% and 8%–11%, respectively. CORT (standard curve 0.05–50 µg/dL) CVs intra- and inter-assay were 4%–6% and 8%–10%, respectively. Peripheral glucose (Glu) and triglyceride (TG) levels were measured using commercial kits (Wiener Laboratory, Rosario, Argentina). 

### 2.3. RPAT SVF Cell and Adipocyte Isolation 

Fresh RPAT pads were dissected, weighed and digested with collagenase as previously reported [29]. Briefly, fat tissues were minced and digested using 1 mg/mL collagenase solution in DMEM (at 37 °C, for 1 h). After centrifugation (at 1000 rpm for 15 min), floating mature adipocytes were separated and reserved for adipocyte size analyses. The SVF cell-pellet was collected, filtered (in a 50-μm mesh nylon cloth) and washed with DMEM (×3). SVF cells were then resuspended in DMEM supplemented with 10% (*vol/vol*) fetal bovine serum (FBS), 4-(2-hydroxyethyl)-1-piperazineethanesulfonic acid (HEPES, 20 nM), 100 IU/mL penicillin and 100 μg/mL streptomycin.

### 2.4. Adipocyte Size Analysis

The size of the isolated fat cells was measured as previously described [30], with minor changes. Briefly, a 50–150-µL aliquot from the top layer obtained as previously described (Section 2.3) was added to 450 µL DMEM. Five to ten microliters from the cell suspension were placed into the Neubauer chamber and coverslipped. Five representative pictures from each sample were taken using a Nikon Eclipse 50i microscope equipped with a camera (Nikon Digital Sight D5-U3, Melville, NY, USA). Cell diameters were measured with image analysis software (Image ProPlus6.0, Rockville, MD, USA). Values below 25 µm were discarded as they can be considered lipid droplets. The values were recorded and assigned to groups differing by 10 µm in diameter, creating a histogram with 10-µm bins. Histograms were used to determine whether the distribution of adipocyte diameters was normal or binomial and to assess the presence of different sized adipocyte subpopulations. We measured an average of 500–600 cells per site to calculate average adipocyte size.

### 2.5. SVF Cell Composition Analysis by Flow Cytometry

SVF cells from RPAT pads from CTR and MSG animals were isolated, and at least 2 × 10^5^ cells (in 100 µL Phosphate-buffered saline (PBS)/0.5% Bovine Serum Albumin (BSA)) were incubated with fluorescent antibodies or respective isotype controls (1/50 diluted, for 1 h at 4 °C). After washing steps, flow cytometry was analyzed using a FACSCalibur flow cytometer (Becton Dickinson Biosciences, San Jose, CA, USA). A combination of cell surface markers was used to identify APCs as: CD34^+^/CD45^−^/CD31^−^ [31]. The conjugated monoclonal antibodies used were: anti-rat CD34:PE (PE: phycoerythrin), anti-rat CD45:FITC (FITC: fluorescein isothiocyanate) and anti-rat CD31:FITC (1 µg/1 × 10^6^ cells, Santa Cruz Biotechnology Inc., Santa Cruz, CA, USA). Samples were analyzed using CellQuest Pro (Becton-Dickinson, San Jose, CA, USA) and FlowJo software (TreeStar, San Carlo, CA, USA).

### 2.6. RPAT SVF Cell Culture and Proliferation

RPAT SVF cells from CTR and MSG groups were seeded (2 × 10^4^ cells/cm^2^) in 24-well plates (Greiner Bio-One, Kremsmünster, Austria) and cultured in DMEM supplemented with 10% (v/v) FBS and antibiotics at 37 °C in a 5% CO_2_-atmosphere [29]. Cells were left in culture to proliferate for up to 9 days (proliferation day: Pd 9). Every 24 h, cells (4 wells per day) were washed (×1) with PBS buffer, and 0.25% (w/v) trypsin solution (dissolved in PBS-EDTA) was added for 2–3 min at 37 °C; the cell suspension was then collected, and the cell number was determined in a Neubauer chamber.

### 2.7. Cell Differentiation Assay

Proliferating CTR and MSG SVF cells (having reached 70%–80% confluence after 5–6 days of culture) were induced to differentiate by the addition of a differentiation mix containing 5 µg/mL INS, 0.25 μM DXM, 0.5 mM 3-isobutyl-l-methylxanthine (IBMX) in DMEM-HEPES, supplemented with 10% FBS and antibiotics [29]. After 48 h, media were removed and replaced with fresh media containing 5 µg/mL INS, 10% FBS and antibiotics (DMEM + INS). Cell samples were harvested on different differentiation days (Dd) and processed for several determinations, as described below. Medium samples of Dd 10 were kept frozen at −20 °C until the measurement of LEP concentrations (Section 2.8.3).

### 2.8. Determinations

#### 2.8.1. RNA Isolation and Real-Time Quantitative PCR

Total RNA was isolated from cells of both groups by the TRIzol extraction method (Invitrogen, Life Tech., Carlsbad, CA, USA). Total RNA was reverse-transcribed using random primers (250 ng) and RevertAid Reverse Transcriptase (200 U/μL, Thermo Scientific, Vilnius, Lithuania). Two microliters of cDNA were amplified with HOT FIRE Pol EvaGreen qPCR Mix Plus (Solis BioDyne, Tartu, Estonia) containing 0.5 μM of each specific primer, using the LightCycler Detection System (MJ Mini Opticon, Bio-Rad, CA, USA). PCR efficiency was near 1. Expression levels were analyzed for β-actin (ACTβ, reporter gene), adiponectin (Adipoq), GR, Leptin (Ob), MR, PPAR-γ2, CCAAT/enhancer binding protein alpha (C/EBPα), Pref-1, Wnt-10b and Zfp423 (the designed primers are shown in alphabetical order in Table 1). Relative changes in the expression level of one specific gene (ΔΔCt) were calculated by the ΔCt method.

#### 2.8.2. Immunofluorescence Assay

CTR and MSG SVF cells were cultured in cover glasses and differentiated as mentioned before. On Dd 4, when it was previously detected that PPARγ2 reaches its highest expression [28], cells were fixed with 10% formalin (for 10–15 min), rinsed twice with PBS and treated with Triton 0.2% for 15 min. Subsequently, cells were incubated overnight with primary antibody PPAR-γ (2 µg/0.1 mL, Santa Cruz Biotechnology Inc., Santa Cruz, CA, USA). For visualization, we used secondary antibody Alexa-Fluor 594 labeled (Invitrogen Life Technologies, Carlsbad, CA, USA), and the nucleus staining was performed with Vectashield (Vector Laboratories, Burlingame, CA, USA) with 4′,6-diamidino-2-phenylindole (DAPI). Immunostaining of the negative control was performed (elimination of primary antibody), which did not show any antiserum immunolabeling. The percentage of PPAR-γ positive cells was expressed in relation to the total number of cells (by counting DAPI stained nucleus, 300–400 total cells per layer at 40× magnification). Immunoreactivity was visualized using a Nikon Eclipse 50i microscope equipped with a Nikon Digital Sight D5–U3 camera (Nikon Instruments Inc., Melville, NY, USA) and NIS-Elements software (Nikon Instruments Inc., Melville, NY, USA). An image editing software program, Adobe Photoshop CS3 (Adobe Systems, San Jose, CA, USA) was used to adjust the contrast and brightness of microphotographs.

#### 2.8.3. Leptin Measurement

Medium LEP concentration was determined by specific RIA developed in our laboratory [32]. In this assay, the standard curve ranged between 0.05 and 25 ng/mL, with coefficients of intra- and inter-assay variation of 4%–7% and 9%–11%, respectively.

#### 2.8.4. Cellular Lipid Content

Cell on Dd 10 were washed with PBS and fixed with 10% formalin (for 10–15 min) in PBS. Then, cells were quickly washed with PBS and stained for 1 h with Oil-Red O (ORO) solution (2:3 *vol/vol* H_2_O:isopropanol, containing 0.5% ORO) [33]. After staining, cells were washed (×3 with PBS), and the dye from lipid droplets was extracted by adding 200 μL isopropanol (10 min). To quantify cell lipid content, sample OD was obtained at 510 nm in a spectrophotometer. Remaining cells were digested with 200 μL 0.25% trypsin solution in PBS-EDTA, at 37 °C for 24 h and centrifuged at 8000× *g* for 15 s. The OD of supernatants was read at 260 nm for DNA quantification, and cell lipid content (measured by ORO and expressed in OD units) was then expressed by the corresponding cell DNA content. 

#### 2.8.5. Cell Differentiation and Maturation

On Dd 10, differentiated cells were fixed with 10% formalin solution for 1 h at room temperature and then stained using the Papanicolaou technique. The percentage of differentiated cells was calculated by counting the total number of cells and that of cells containing lipid droplets, when visualized in a light microscope (after counting 200–250 total cells per layer, at 40× magnification). Lipid-containing cells were assigned to 3 graded stages of maturation according to the nucleus position: stage I (GI, central), stage II (GII, between central and peripheral) and stage III (GIII, completely peripheral) [34]. The percentage of cells corresponding to different maturation stages was expressed in relation to the total number of differentiated cells. Image analysis was assessed using a using a Nikon Eclipse 50i microscope equipped with a camera Nikon Digital Sight D5–U3 (Nikon Instruments Inc., Melville, NY, USA) and image analysis software (Image ProPlus 6.0, Rockville, MD, USA).

### 2.9. Statistical Analysis

Results are expressed as mean values ± SEM. Data were analyzed by ANOVA (one-way) followed by Fisher’s test. To determine the differential effect of the treatment according to age, ANOVA (two-way) followed by the Bonferroni post-test were performed. For comparison of adipocyte size populations between groups, the non-parametric Mann–Whitney test was used. The normal or binomial distribution of adipocyte size data was determined by the Kruskal–Wallis test, followed by the Mann–Whitney test. In all cases, *p*-values lower than 0.05 were considered statistically significant. All statistical tests were performed using GraphPad Prism 6.0 (GraphPad Software Inc., San Diego, CA, USA). 

## 3. Results

### 3.1. The MSG Rat Phenotype

**Figure 1:**
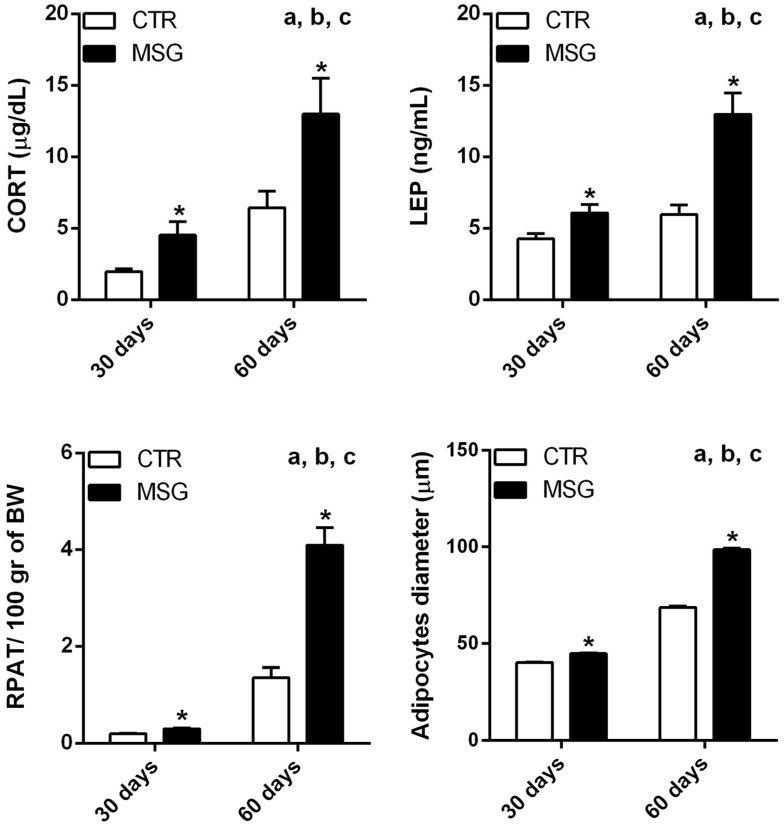
Comparison of hormone levels and retroperitoneal adipose tissue (RPAT) parameters from CTR and MSG rats. Corticosterone (CORT) and leptin (LEP) plasma levels; RPAT/100 g of BW and RPAT adipocyte size from rats at 30 and 60 days of age. Values are means ± SEM. * *p* < 0.05 vs. CTR values for similar ages. Two-way ANOVA: ^a^ those parameters significantly affected by age; ^b^ those significantly affected by treatment; ^c^ a significant synergic effect of MSG treatment and age.

Thirty-day-old MSG rats displayed lower BW (Table 2) and significantly higher LEP and CORT levels, as well as increased RPAT adipocyte diameter and RPAT/100 g of BW (Figure 1). Glu, INS and TG plasmatic levels remained similar to CTR litter-mate values (Table 2). As we have previously shown [28], 60-day-old MSG rats showed the same altered parameters as 30-day-old MSG rats (Table 2 and Figure 1), but when we compared all of these parameters in both ages, we found that the circulating levels of LEP and CORT, as well as RPAT/100 g of BW and RPAT adipocyte hypertrophy increased in MSG rats during development (Figure 1). 

### 3.2. Proliferation Capacity of SVF Cells from MSG Rats at 30 Days of Age

After seeding, the proliferation capacity of RPAT SVF cells from both groups was assessed by counting the cell number every 24 h and recording those numbers throughout the proliferation period (Pd 1–Pd 9). Data indicated a significant (*p* < 0.05) decrease in the proliferation capacity of cells from the MSG group, which was noticed at the end of the proliferation period (158,083 ± 13,086 and 103,312 ± 17,028 cells/well on Pd 8 and 177,020 ± 11,097 and 115,688 ± 15,433 cells/well on Pd 9, in CTR and MSG groups, respectively). Moreover, the slopes of these curves were as follows: 21,722 ± 1457 and 16,628 ± 2055 cells per day, in CTR and MSG cell groups, respectively (*p* < 0.05).

### 3.3. Enhanced Terminal Differentiation of SVF Cells from MSG Rats at 30 Days of Age

**Figure 2:**
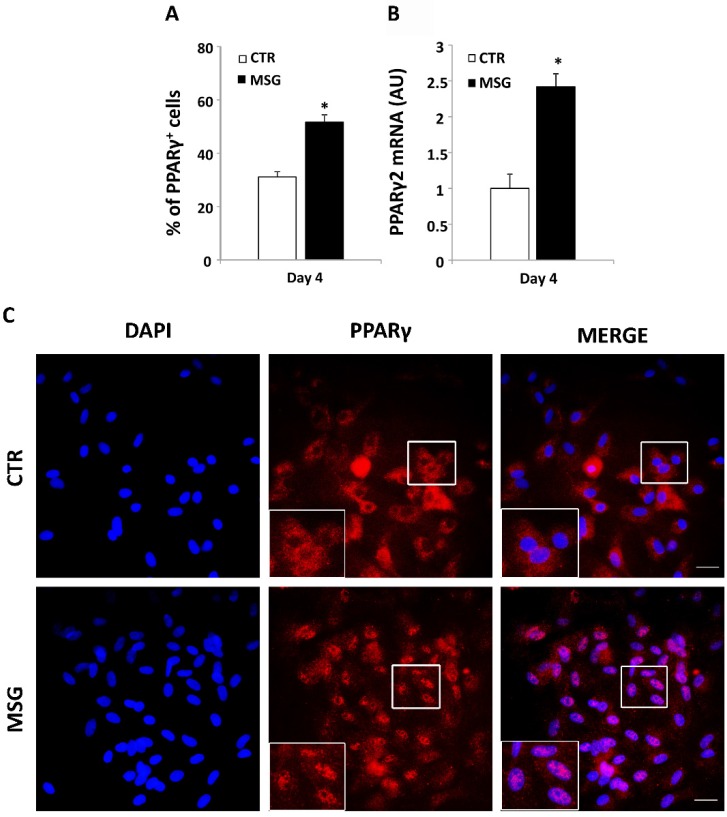
MSG cells display higher expression of Peroxisome proliferator-activated receptor (PPAR)γ2 during in vitro differentiation. (**A**) Percentage of PPARγ positive cells related to the total cell number counted by nucleus 4′,6-diamidino-2-phenylindole (DAPI) staining; (**B**) mRNA cell expression levels of PPARγ2 in CTR and MSG stromal vascular fraction (SVF) cells on Differentiation Day (Dd) 4 (AU: arbitrary units). Values are the means ± SEM (*n* = 5/6 different experiments). * *p* < 0.05 vs. CTR values; (**C**) Representative images of DAPI nucleus staining (blue), PPARγ positive cells (red) and merged image of CTR and MSG cells on Dd 4 (40× magnification, scale bar at 20 μm). The insert shows cytoplasmatic or nuclear PPARγ localization.

**Figure 3:**
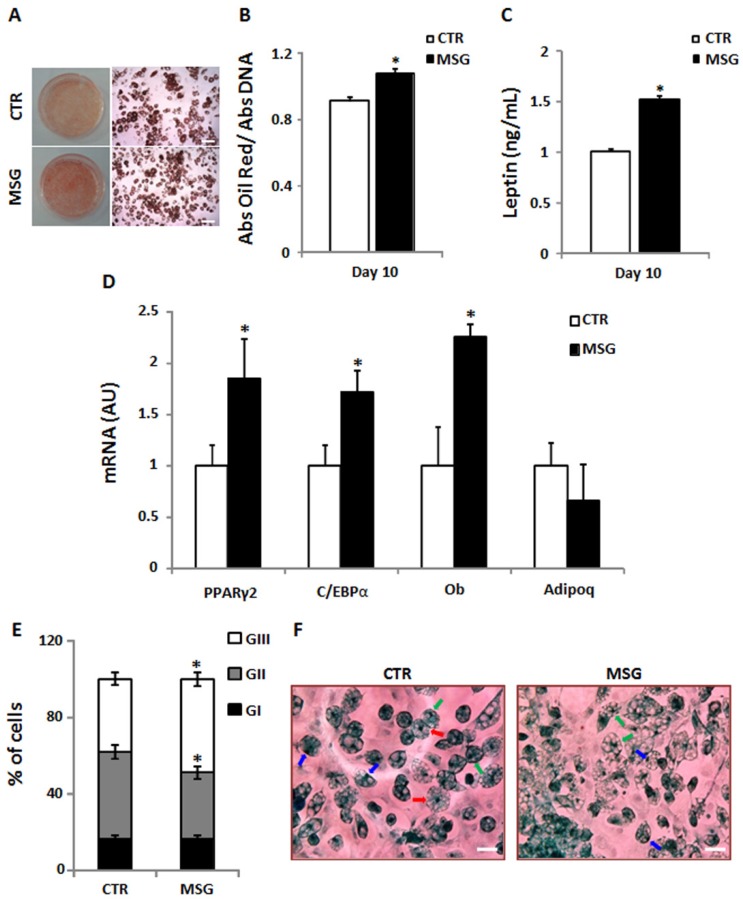
MSG cells exhibit increased in vitro adipocyte differentiation. (**A**) Macroscopic (left panel) and microscopic (right panel, magnification 10×, scale bars at 200 µm) views of the Oil Red O-stained dishes; (**B**) Quantification of intracellular lipid accumulation; (**C**) cell leptin secretion (*n* = 5/6 different experiments with 10/12 wells per day per experiment) and (**D**) cell mRNA expression levels of PPAR-γ2, CCAAT/enhancer binding protein (C/EBP)α, Leptin (Ob) and Adiponectin (Adipoq) on Differentiation day (Dd) 10 of SVF cells isolated from RPAT pads of 30 day-old CTR and MSG male rats (*n* = 5/6 different experiments) (AU: arbitrary units); (**E**) Percentages of cells according to their stages of maturation; (**F**) Representative fields containing in vitro differentiated CTR and MSG adipocytes (stained on Dd 10, magnification 40×, scale bars at 20 µm), displaying different degrees of maturation depending on the nucleus position: GI, central (red arrow); GII, displaced from the center (green arrow); and GIII: fully peripheral (blue arrow) (*n* = 4/5 different experiments; data from 200/250 cells were recorded in each experiment). Values are means ± SEM *, *p* < 0.05 vs. CTR values.

On Dd 4, MSG cultured SVF cells showed a higher percentage of PPARγ positive cells (Figure 2A) in addition to enhanced PPARγ2 expression on the same culture day (Figure 2B). Intracellular lipid content and cell LEP release into the culture medium on Dd 10 were evaluated as parameters of adipocyte differentiation. As shown in Figure 3, MSG adipocytes displayed significantly (*p* < 0.05 vs. CTR values) higher lipid content (Figure 3A,B) and LEP release (Figure 3C). The expression of marker genes of fully-differentiated cells (on Dd 10), such as Ob, Adipoq, C/EBP-α and PPAR-γ2 mRNAs, were significantly (*p* < 0.05) higher in MSG than in CTR cells; conversely, no difference was noticed in Adipoq mRNA concentration (Figure 3D). Additionally, we determined the extent of adipogenesis in vitro by quantifying the percentage of differentiated cells at the end of the differentiation period (Dd 10). Our results showed no differences among groups for this parameter (53.99% ± 1.90% and 50.29% ± 2.8% of differentiated cells in the CTR and MSG groups, respectively; *n* = 3 independent experiments). However, differences in the percentage of cells at different maturation degrees were found. Indeed, MSG specimens showed a higher percentage of cells at an advanced maturation degree (GII and GIII; see Figure 3E,F), thus indicating that MSG cells maturate faster than CTR cells.

### 3.4. Age-Dependent Effect of GCs on APCs’ Competency and Adipogenic Potential 

**Figure 4:**
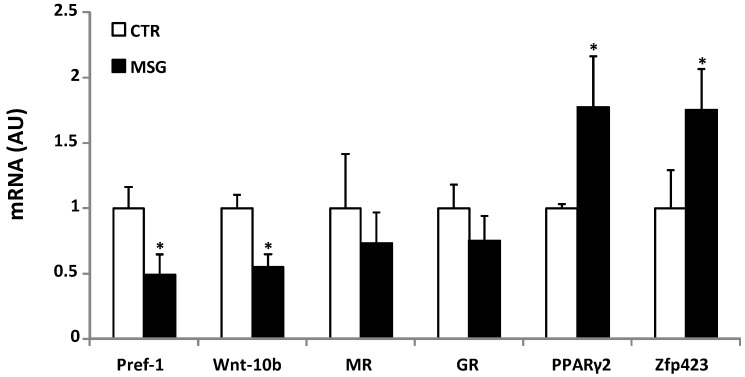
MSG SVF cells displayed higher competency, but lower anti-adipogenic factors expression. RPAT-SVF cell mRNA levels of anti-adipogenic (Pref-1 and Wnt-10b), glucocorticoid and mineralocorticoid receptors (GR and MR) and competency markers (PPAR-γ2 and Zfp423) from CTR and MSG male rats (AU: arbitrary units).Values are means ± SEM (*n* = 5/6 different experiments). * *p* < 0.05 vs. CTR values.

Taking into account the differences found in the adipogenic capacity of MSG cells, the expression levels of several pro- and anti-adipogenic factors expressed by SVF cells were analyzed. MSG RPAT SVF cells expressed significantly (*p* < 0.05) lower levels of Pref-1 and Wnt-10b (Figure 4), whereas the expression of MR and GR genes remained the same. These results are different from those previously found for SVF cells from MSG rats at 60 days of age, where anti-adipogenic factors showed higher expression and the MR mRNA level was lower [28]. Similarly, the expression levels of Zfp423 and PPARγ2 were higher in SVF cells from MSG rats at 30 days of age (Figure 4), in contrast with the corresponding values previously found for SVF cells from MSG rats at 60 days of age [28]. Taken together, these data clearly indicate that high GC plasmatic levels induce a different effect on APCs’ adipogenic competency, a fact probably dependent on the differential cell pattern of MR gene expression.

### 3.5. High Peripheral GC Levels Do Not Modify APC Number over Development

**Figure 5:**
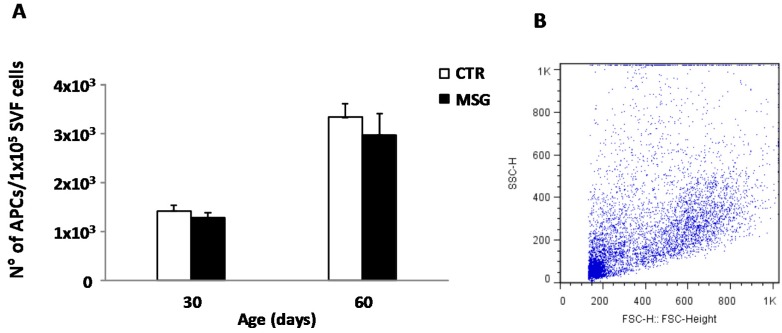

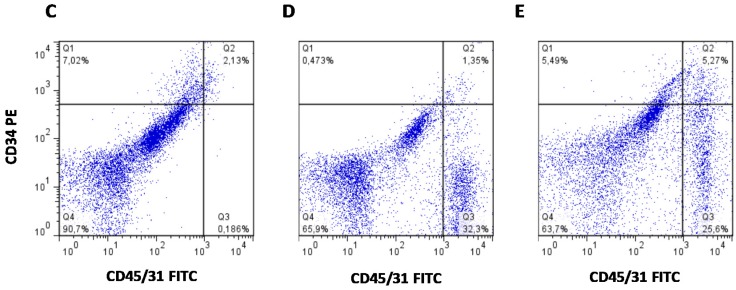
High GC exposure does not change adipocyte precursor cells (APCs) number. (**A**) Number of APCs in SVF from CTR and MSG animals at both ages. Adipocyte progenitors were identified by flow cytometry analysis using the CD34^+^/CD31^−^/CD45^−^ profile; (**B**) Characteristic forward versus side scatter dot plot of freshly-isolated SVF cells from RPAT. Fluorescence profiles obtained for (**C**) IgG isotype control FITC conjugated combined with CD34 PE staining; (**D**) IgG1 isotype control PE conjugated combined with CD45/CD31 FITC staining and (**E**) APC subset (upper left quadrant). FITC: fluorescein isothiocyanate; PE: phycoerythrin. Values are means ± SEM (*n* = 3/4 different experiments).

The adipogenic population (CD34^+^/CD31^−^/CD45^−^) contained in the RPAT SVF from 30- and 60-day-old rats from both groups was evaluated in order to assess a possible GC effect on APC number. Interestingly, GCs exposure did not change the APC number at any age studied (Figure 5). These data strongly suggest that the main GC effect on SFV cells is on APCs’ competency rather than on cell number. 

### 3.6. Relationship between APCs’ Competency and Adipogenic Capacity in MSG Rats

**Figure 6:**
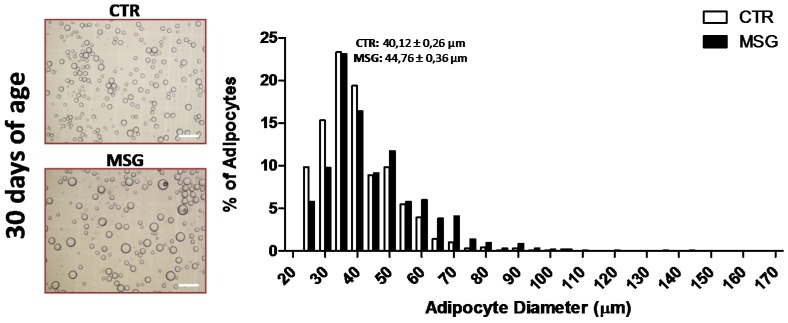

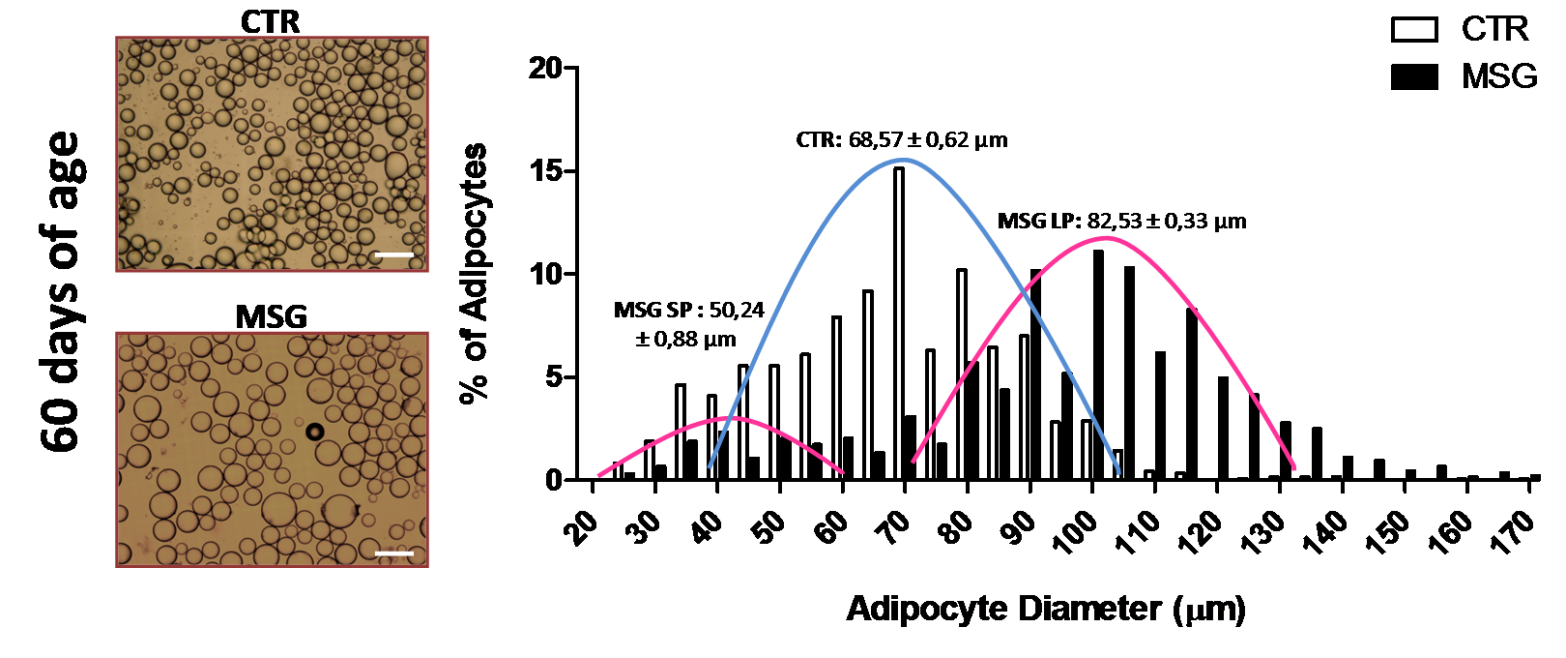
In vivo adipogenesis occurs in MSG animals. Adipocyte size distribution in RPAT depot from CTR and MSG rats at 30 (upper panel) and 60 days of age (lower panel). Purple and blue lines represent MSG (MSG SP: MSG small population; MSG LP: MSG large population) and CTR adipocyte populations, respectively. Representative images of mature adipocytes in cell suspension are shown for each group (magnification 10×, scale bars at 200 µm). Values are the means ± SEM (*n* = 3 animals per group).

As described above, APCs from MSG rats at 30 days of age displayed an increased competency and differentiated rapidly when compared to CTR cells. Therefore, in order to establish any possible relationship between these data and the characteristic of in vivo RPAT mass expansion, the size distribution of mature adipocytes contained in RPAT pads from CTR and MSG rats of both age groups (Figure 6) was determined. Our data indicate that adipocytes from 30-day-old MSG animals were hypertrophic vs. age-matched CTR cells (Figure 6, upper panel). However, the smaller size of all adipocytes from 30-day-old rats compared to those from 60-day-old rats suggests that newly-generated adipocytes appeared at 30 days of age, regardless of the group examined. Interestingly, the profile of adipocyte size found in RPAT pads from 60-day-old MSG rats was conspicuous. In fact, while two adipocyte populations were found in the MSG group, one smaller and the other larger, a unique adipocyte size (intermediate) population characterizes CTR RPAT pads (Figure 6, lower panel). These data reveal that the in vivo activation of the adipogenic process could be dependent on a higher APCs competency in the 30-day-old MSG rat. 

## 4. Discussion

Enhanced VAT is associated with an increased risk of the development of several pathologies, such as diabetes mellitus type 2 and cardiovascular disease. Conversely, SCAT function is considered as protective against them [35,36]. This difference has been attributed, at least in part, to a higher adipogenic capacity of SCAT [37]. However, a system has been recently developed for the inducible and permanent labelling of mature adipocytes in vivo, which indicates that hyperplastic expansion of the AT occurs mainly at the epididymal, but not at the SCAT depot [38]. The present work showed that high chronic GC plasma levels are associated with early activation of adipogenesis (30-day-old MSG rats), supporting the concept that RPAT could expand through the generation of new adipocytes. In contrast, our previous studies revealed the inhibition of the RPAT adipogenic process at advanced age (60 day-old MSG rats) [28]. This age-/time-dependent shift in the adipogenic potential could be a consequence of changes in the expression levels of APCs’ competency factors.

GCs have diverse effects on both AT functions [39,40] and distribution [41,42], favoring an increase in VAT, but not in SCAT mass. MSG rats share several features of the human CS phenotype, which is characterized by chronic high circulating levels of GC, enhanced AT mass and adipocyte size and an altered pattern of adipokine secretion, among others. In the MSG rat phenotype, restoring to normal the peripheral levels of GC reverses most of such dysfunctions [43]. Our present data indicate that the MSG animal phenotype is already established at 30 days of age, and some characteristic alterations get differentially worse over time. 

Regarding the terminal stage of adipogenesis, an increase in all differentiation parameters was observed in MSG cells from 30-day-old rats. According to these findings, the expression of anti-adipogenic factors, such as Pref-1 and Wnt-10b decreased in undifferentiated MSG cells. In fact, the pro-adipogenic action of GCs is due to its inhibitory effect on Pref-1 and Wnt/b-catenin signaling pathway expression [21]. As mentioned above, these results show evidence of a dual behavior of the adipogenic process by itself in a GC-rich endogenous environment, characterized by adipogenesis activation at an initial step and the subsequent inhibition of the process, as shown before [28].

Cristancho et al. [15] distinguished between two functional terms: cell competency and cell commitment, where adipogenic competency refers to the cell’s ability to differentiate into adipocyte upon the addition of defined stimuli, and adipogenic commitment indicates the multipotent cell type fate to undergo its conversion into adipocyte. Within this context, the adipogenic potential of an AT pad depends, besides any adipogenic stimulus environment, on the number and competency of APCs present in the local SVF. GCs are known to inhibit the proliferation capacity of APCs and to play an important role driving APCs to differentiate into adipocytes.

Over the last few years, great effort has been made to find cell surface markers that unequivocally identify APCs applying diverse criteria. Most of them agree that the CD34 antigen can distinguish between cells that are able or not to differentiate into mature adipocytes [44,45,46]. Interestingly, the expansion of different AT pads, both in mouse and human obese phenotypes, has been proposed to depend on changes in progenitor cell number [10,37,47]. To our knowledge, the effect of chronic exposure to high GC levels on APCs number in VAT pad has not been yet explored. In fact, our study is the first to show that APCs number in the SVF of RPAT from MSG rats is not altered at 30 or 60 days of age, and consequently, this parameter does not seem to be responsible for the changes observed in their adipogenic potential.

Zfp423 has been described as a marker of APCs competency, because it regulates PPARγ2 expression throughout the amplification of SMAD protein activity [17]. Thus, since PPARγ2 plays a key role within the overall adipogenic process, we are persuaded that PPARγ2 should be considered as a competency factor by itself. In SVF cells from 30 day-old MSG rats, the levels of PPARγ2 and Zfp423 mRNA were higher than in CTR SVF cells, which indicates high APC competency. These data are opposite to those found in 60 day-old MSG rat cells where both PPARγ2 and Zfp423 mRNA levels were low. Our results demonstrate that changes in APCs competency are responsible for the different adipogenic capacities of SVF cells from MSG rats of 30 and 60 days of age. 

In obese phenotypes, a recruitment of immune cells occurs in VAT, including macrophages, lymphocytes and neutrophils, thus contributing to the development of a chronic inflammatory state [48,49,50,51]. Furthermore, there is a macrophage polarization toward the M1 type, which secretes pro-inflammatory cytokines (e.g., TNFα, IL1β and MCP-1) to the detriment of the anti-inflammatory M2 type (IL-10) [52,53]. These pro-inflammatory macrophage-secreted factors have been involved in an antiadipogenic activity, by decreasing the expression levels of adipogenic markers, such as aP2 and PPARγ2 in human and 3T3-L1 preadipocytes [54,55]. The role of GCs mediating the inflammatory response in AT depends on the MR or GR activation, which will determine a pro- or anti-inflammatory response, respectively, due to the decrease or increase of pro-inflammatory cytokines’ secretion [26]. Nevertheless, CS patients display increased adiposity, but the establishment of a chronic inflammatory state is controversial [56,57]. We have previously shown that RPAT expression of TNFα, IL-6, MCP-1 and F4/80 does not increase in 60-day-old MSG rats, probably due to the anti-inflammatory effect of GCs exerted through GR activation [28]. In this regard, further studies are needed to evaluate the AT inflammatory response in MSG rats at 30 days of age.

Pro-adipogenic activity of GCs has been clearly recognized; then, the co-existence of high GC levels and a reduced adipogenic cell capacity could be indicative of changes in the RPAT sensitivity to GCs. This could explain why high levels of a potent adipogenic factor do not stimulate the process continuously, preventing hypertrophy of VAT. It is known that aldosterone is able to promote differentiation of APCs in both 3T3-L1 [58] and human preadipocytes. Notably, expression silencing or activity blocking of MR in both cell types prevent adipogenesis, but this is not the same for GR inactivation [25,26]. We found that MR mRNA levels did not change in 30-day-old MSG rats, though they decreased in 60-day-old rats, coinciding with the low adipogenic capacity found in them [28]. This could suggest that MR is involved in the development of a GC-resistant state, although the participation of MR or GR in the biological actions of GCs upon the AT is still being debated [25,26,27]. However, the contribution of GR to the lack of GC effect cannot be ruled out, despite not finding any change in cell GR expression.

The distribution of adipocyte size has been previously used to determine the presence of AT hypertrophy and/or hyperplasia [10]. Analysis of the adipocyte size distribution in RPAT from MSG animals at both ages showed interesting data. Indeed, MSG rats at 30 days of age displayed one hypertrophic adipocyte population, while MSG rats at 60 days of age presented two adipocyte size populations, one smaller and another larger than the only adipocyte size population found in CTR pads. The additional presence of small adipocytes in 60-day-old MSG rats suggests that increased APC competency (seen in 30-day-old MSG rats) leads to adipogenesis stimulation between Days 30 and 60 of age.

## 5. Conclusions

The present study shows that changes in competency and/or number of APCs are two independent mechanisms that can modulate the adipogenic potential of an AT depot. In particular, long term in vivo cell exposure to high GC levels induces an early stimulation and subsequent inhibition of RPAT adipogenesis, a dual age-dependent process, mainly due to changes in APCs’ competency, but not in number. Metabolic dysfunctions associated with obesity are dependent on the development of adipocyte hypertrophy; hence, the possibility of increased APC competency and/or number could result in the activation of the adipogenic process by activating hyperplastic VAT expansion, with consequent benefits for health.

## Figures and Tables

**Table 1:** Primers used for real-time polymerase chain reaction (PCR) analysis.

	Primers (5′-3′)	GBAN	bp
β-actin (ACTβ)	se, AGCCATGTACGTAGCCATCC	NM_031144	115
	as, ACCCTCATAGATGGGCACAG		
Adiponectin (Adipoq)	se, AATCCTGCCCAGTCATGAAG	NM_144744	159
	as, TCTCCAGGAGTGCCATCTCT		
CCAAT/enhancer binding protein alpha (C/EBPα)	se, CTGCGAGCACGAGACGTCTATAG	NM_012524	159
	as, TCCCGGGTAGTCAAAGTCACC		
Glucocorticoid Receptor (GR)	se, TGCCCAGCATGCCGCTATCG	NW_047512	170
	as, GGGGTGAGCTGTGGTAATGCTGC		
Mineralocorticoid Receptor (MR)	se, TCGCTCCGACCAAGGAGCCA	NM_013131	193
	as, TTCGCTGCCAGGCGGTTGAG		
Leptin (Ob)	se, GAGACCTCCTCCATCTGCTG	NM_013076	192
	as, CTCAGCATTCAGGGCTAAGG		
Peroxisome proliferator-activated receptor gamma 2 (PPAR-γ2)	se, AGGGGCCTGGACCTCTGCTG	NW_047696	185
	as, TCCGAAGTTGGTGGGCCAGA		
Preadipocyte Factor 1 (Pref-1)	se, TGCTCCTGCTGGCTTTCGGC	NM_053744	113
	as, CCAGCCAGGCTCACACCTGC		
Wingless-type MMTV integration site family member 10b (Wnt-10b)	se, AGGGGCTGCACATCGCCGTTC	NW_047784	175
	as, ACTGCGTGCATGACACCAGCAG		
Zinc finger protein 423 (Zfp423)	se, CCGCGATCGGTGAAAGTTG	NM_053583.2	121
	as, CACGGCTGGATTTCCGATCA		

Rat-specific primers for real-time PCR are listed in alphabetical order. se: sense; as: anti-sense; GBAN: GenBank Accession Number; amplicon length in bp.

**Table 2:** Metabolic parameters of control (CTR) and Monosodium l-glutamate (MSG) treated rats.

	CTR 30-Day-Old	MSG 30-Day-Old	CTR 60-Day-Old	MSG 60-Day-Old
Body Weight (BW, g)	78.84 ± 1.78	71.23 ± 1.56 *	312.34 ± 9.32 ^a,b,c^	242.50 ± 5.47 *^,a,b,c^
Insulin (INS, ng/mL)	0.25 ± 0.02	0.31 ± 0.04	2.01 ± 0.05 ^a^	2.42 ± 0.35 ^a^
Glucose (Glu, g/L)	1.26 ± 0.03	1.27 ± 0.03	1.17 ± 0.03	1.14 ± 0.06
Triglyceride (TG, g/L)	0.82 ± 0.06	1.19 ± 0.11	1.16 ± 0.14	1.24 ± 0.22

BW and plasma levels of INS, Glu and TG in CTR and MSG rats at 30 (*n* = 20 rats per group) and 60 days of age (*n* = 10 rats per group). Values are the means ± SEM. * *p* < 0.05 vs. CTR values of similar age. Two-way ANOVA: ^a^ those parameters significantly affected by age; ^b^ those parameters significantly affected by treatment; ^c^ a significant synergic effect of MSG treatment and age.
